# Community mental health services in Southern Gauteng: An audit using Gauteng District Health Information Systems data

**DOI:** 10.4102/sajpsychiatry.v23i0.1055

**Published:** 2017-07-05

**Authors:** Lesley J. Robertson, Christopher P. Szabo

**Affiliations:** 1 Department of Psychiatry, School of Clinical Medicine, University of the Witwatersrand, South Africa; 2 Sedibeng District Health Services, South Africa; 3 Department of Psychiatry, Charlotte Maxeke Johannesburg Academic Hospital, South Africa

## Abstract

**Background:**

Community mental health services (CMHS) are a central objective of the National Mental Health Policy Framework and Strategic Plan. Three core components are described: residential facilities, day care and outpatient services. Primary mental health care with specialist support is required according to an intervention pyramid. Staffing norms provide for a minimum mental health service coverage of 2.7% of the population for adults and 1.5% for children and adolescents.

**Aim:**

The aim of this study was to describe the existing CMHS in Southern Gauteng in terms of the National Mental Health Policy.

**Methods:**

The CMHS of the City of Johannesburg, Ekurhuleni, Sedibeng and West Rand districts were studied. Information regarding service organisation and staffing was obtained via the Gauteng Directorate of Mental Health. Routinely collected District Health Information Systems data for the 2014/2015 year were analysed.

**Results:**

The organisation of services was not consistent with that recommended by the Mental Health Policy, and specialist CMHS were inappropriately situated within primary care. Only 2.23% of clinic visits were for mental health, and 80% of these were at specialist CMHS. Overall mental health coverage was approximately 0.3% of the population for adults and 0.02% for children and adolescents. Staffing, residential facilities and day care were far below the cited norms for minimal cover.

**Conclusion:**

Our audit revealed that the CMHS in Southern Gauteng did not meet any of the norms cited by the Mental Health Policy. Barriers to implementation of this aspect of the Mental Health Policy need to be explored.

## Introduction

In South Africa, community-based mental health care is a requirement of the Mental Health Care Act of 2002^1^ and a central objective of the National Mental Health Policy Framework and Strategic Plan 2013–2020 (MH Policy).^2^ Advantages of community mental health services (CMHS) over psychiatric hospital–based care lie not only in that they meet the legal and human rights of mental health care users (MHCUs) to receive care close to home but also in their modelled cost-effectiveness in terms of improved population coverage.^3,4^ Three core components are listed on page 23 of the MH Policy: community residential facilities, day care and outpatient services. The bulk of care should be provided by primary health care (PHC) practitioners, with specialist supervision and care for MHCUs with more complex conditions requiring specialised assessment and/or intervention.^3^ The MH Policy positions the specialist CMHS back-to-back with general hospital acute psychiatric units within an intervention pyramid. They are tasked with providing continuity of care for the severely ill after hospital discharge, facilitation of hospital referrals, supervision of PHC, community outreach, and engagement with non-health sectors such as the South African Police Service, local schools and non-governmental organisations. Areas for strengthening district health services are also identified within the MH Policy, and modelled norms and standards for both adult and child and adolescent CMHS are referenced.^2,3,5,6,7^

In contrast to the MH Policy, the National Health Strategic Plan 2014 and 2015 – 2018 and 2019^8^ and the white paper for National Health Insurance (NHI)^9^ make no provision for CMHS. Whilst PHC re-engineering to provide integrated primary mental health care is endorsed, specialist services are reserved for general regional and tertiary hospitals and specialised psychiatric hospitals. This is despite the deinstitutionalisation process that has taken place in South Africa over the past two decades. Nevertheless, CMHS do exist in those areas of Southern Gauteng served by the Department of Psychiatry of the University of the Witwatersrand. These services developed gradually, beginning in the 1990s, in response to the need to provide specialist community care to deinstitutionalised MHCUs. Of note is that the CMHS were not driven by policy or cohesive planning. They grew in an ad hoc manner, at the request of psychiatrists and the discretion of each District Health Director. Academic mental health specialists were appointed to the primary care mental health programme within the district health administration and budget. For monitoring and evaluation, the CMHS were included as secondary level care mental health clinics in the routine data collected by the provincial District Health Information Systems (DHIS).^10^ These data are available to Gauteng Health employees and are useful in that they provide a broad overview of the CMHS.

A description of the Southern Gauteng CMHS, together with a list of most of the clinics and an audit of the staffing and patient numbers served was previously conducted in 2005.^11^ No assessment of the service has been published since then. With the development of norms and standards, the promulgation of the MH policy, the implementation of PHC re-engineering and the prospect of NHI, it seems relevant to assess the current situation using the data available for provincial planning.

## Aim

Our aim was to describe the CMHS in Southern Gauteng in terms of the structure and norms proposed by the MH Policy. The primary objective was to gain better understanding of the current situation to advance the implementation of policy.

## Methodology

### Study design and setting

A retrospective secondary analysis of the DHIS data collected for the 2014 and 2015 financial year was performed. Additional information regarding the organisational structure and staffing of the CMHS and hospital psychiatric units was obtained from the Gauteng Directorates of Mental Health and of Specialised Services, district mental health managers and district psychiatrists.

The DHIS collected data in the form of clinic visits. The data were captured by the DHIS from tick-box forms completed on site by clinic staff. We studied the cleaned data for the Gauteng region served by the University of the Witwatersrand, that is, the two metropolitan areas of City of Johannesburg (COJ) and Ekurhuleni, and the two districts of Sedibeng and West Rand. Over 9 million people are served by the CMHS in these districts (Table 1).^12^ On average, 24% are under the age of 15 years and 4% over 65 years.

**TABLE 1:** District population indicators, according to the National Census, 2011.

National Census 2011 Population Indicator	Southern Gauteng	City of Johannesburg	Ekurhuleni	Sedibeng	West Rand
General population, 2011	9 350 776	4 434 827	3 178 470	916 484	820 995
General population, 2001	7 246 532	3 226 055	2 481 762	794 088	744 627
% Population growth per annum, 2001–2011	2.68	3.18	2.47	1.43	0.98
% Increase in population, 2001–2011	29.0	37.5	28.0	15.4	10.3
Unemployment rate (%)	26.3†	25.0	28.8	31.9	26.3
Youth (15–34 years) unemployment rate (%)	34.0†	31.5	36.9	41.7	35.2
Matriculate rate of adults ≥20 years (%)	34.7†	35.0	35.9	32.7	30.7
Housing: % population with formal dwellings	79.6	81.4	77.4	84.8	72.7

*Source*: Statistics South Africa 2012

† Figures for the whole of Gauteng, including Tshwane.

### Data sample and analysis

Data from a total of 301 district clinics were available for the period 01 April 2014 – 31 March 2015. The sample selected for the study was of the data collected for the following indicators:

1. At PHC level clinics:
  - Total PHC visits.
  - First visit for mental illness.
  - Follow up visit for mental illness.
2. At secondary care level mental health clinics (CMHS):
  - Mental health visit by people aged 18 years or more.
  - Mental health visit by people under the age of 18 years.
  - Referral in, from:
    ▪ Self (includes MHCUs brought in by relatives or friends).
    ▪ PHC.
    ▪ Hospital (includes from medical or psychiatric units).
    ▪ Other sector (includes any health or non-health sectors, e.g. schools).
  - Referral out, to:
    ▪ PHC.
    ▪ Hospital (includes any hospital).

The other health indicators were excluded as they did not contribute any additional information. The data were analysed on Excel^®^ using descriptive statistics.

### Study tools

#### National Mental Health Policy Framework and Strategic Plan

The following modelled norms and standards referenced by the MH Policy were used for comparison:

1. **Organisation of services:**^2^ The intervention pyramid and accompanying description of services on pages 22–24 of the MH Policy.
2. **The human resource norms required for a minimal service level population coverage:**^3^ The minimal service cover takes into account that South Africa is not able to afford CMHS for all individuals with mental illness, and, as revealed by the South African Stress and Health (SASH) study,^13^ that less than a third of those with common mental illness seek help from formal mental health services. Minimal service level aims to serve 50.0% of people with schizophrenia and bipolar disorder and 30.0% of those with common mental illness. Calculated according to the estimated prevalence rates of different types of mental disorders and adjusting for comorbidity, this equates to 2.7% of the general population. The proposed human resource norms for adult mental health care are based on likely service utilisation, and staffing needs to serve 2.7% of a hypothetical population of 100 000 people.
  Regarding child and adolescent mental health services, minimal coverage aims to serve between 15.0% and 30.0% of those with mental illness and equates to 1.5% of the general population.^6^ Although separate human resources are proposed, these were not used in this study as the same CMHS provided child and adolescent as well as adult mental health care.
3. **Residential and day care facilities for people with mental illness:**^3^ For adults, 107 residential beds and 194 day care places per 100 000 population are recommended.
4. **Balance between CMHS and hospital psychiatric care:**^5^ In the norms modelled for the care of adults with severe psychiatric conditions, a number of beds per 100 000 population is proposed for hospital care (acute and medium-long stay beds) and for community residential care. The necessary staffing to support the proposed hospital bed numbers and community residential and ambulatory care is also modelled. In this model, a target community: hospital staffing ratio of 1:2 is proposed. For this study, the ratio was only applied to the number of psychiatrists in community and hospital-based care.

## Ethical consideration

Permission to use the DHIS data in a peer-reviewed publication was granted by the Gauteng Directorate of Policy, Planning, Research and Monitoring and Evaluation. The study was approved by the Health Research Ethics Committee of the University of the Witwatersrand

## Results

### Organisation of the community mental health services

The CMHS were administered by the District Health Services within the PHC budget. They consisted of an outpatient service operating from the PHC clinics and of residential and day care facilities. Psychopharmacological care was provided by psychiatric registrars and medical officers under the supervision of consultant psychiatrists. Most MHCUs collected repeat prescriptions from the CMHS nursing staff; more stable users would collect from PHC nurses and return to the CMHS for medical review. Psychotherapeutic services were provided by psychologists attached to the CMHS. With PHC re-engineering, there were no occupational therapists or social workers attached to the CMHS; MHCUs requiring these services were referred to the generalist PHC occupational therapists and social workers, who were not required to have any specialist mental health expertise.

Specialist level medications were available in each district according to the National Essential Medicines List. Nursing staff were responsible for issuing of repeat medication, monthly review of the mental state and well-being of the MHCUs, co-ordination of hospital and inter-sectoral referrals, psychoeducation of the patients and their families, and conducting local mental health awareness campaigns. However, there was no consistent system of assertive psychiatric nursing with active tracing of non-adherent MHCUs or home visits.

The CMHS functioned independently of the general hospital acute psychiatric units, which fall under Hospital Services administratively. There were no designated case managers for the co-ordination of patient care between hospital and district. PHC re-engineering towards an integrated model of chronic disease management had begun in all districts. Training of PHC practitioners in primary mental health care was provided by the CMHS psychiatrists and nursing staff. However, although ward based PHC outreach teams and community health workers had been introduced in all areas, mental health was not included in the training manuals.

### Clinic visits and population coverage

**FIGURE 1:**
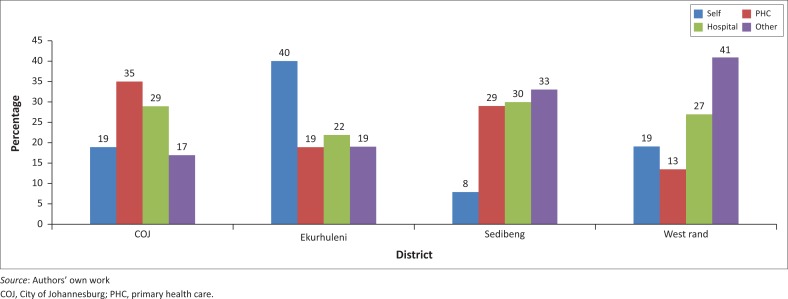
Referral source for the Community Mental Health Services of each district.

A total of 18 751 326 clinic visits were recorded during the 2014 and 2015 financial year, of which 2.23% (428 844 visits) were for mental health. However, primary mental health care accounted for only 0.5% of all clinic visits, as 80% of the mental health visits were attended by the specialist staffed CMHS and only 20% by PHC. Regarding the referral of MHCUs to the CMHS, about 25% of MHCUs were referred to the CMHS from each referral source, although the proportions differed with each district (Figure 1). Very few MHCUs were referred out from the CMHS to PHC. The total numbers for the year were 366 in COJ, 275 in Ekurhuleni, 1151 in Sedibeng and 87 in West Rand. Regarding both PHC mental health visits and referrals from CMHS to PHC, the data did not reveal how many of these only attended PHC for the collection of medicine, with clinical review and repeat prescription occurring at the CMHS. This is important as the latter group would not constitute true primary mental health care.

As, in general, the MHCUs attending the CMHS are chronic care users who visit the clinic on a monthly basis, the monthly average should correspond roughly to the number of MHCUs served. Therefore, it may be estimated that just under 27 000 adults and almost 2000 children and adolescents were attended to by the CMHS over the 12-month period (Table 2). This equates to approximately 0.3% of the general population for adults and 0.02% of the general population for children and adolescents, far below the 2.7% and 1.5% recommended for minimal coverage.^3,6^

**TABLE 2:** Estimated number of mental health care users attending the Community Mental Health Services with respect to population.

Variable	City of Johannesburg	Ekurhuleni	Sedibeng	West Rand	Total
General population^12^	4 434 827	3 178 470	916 484	820 995	9 350 776
Target MHCUs ≥ 18 years (2.7% of population)^3^	119 740	85 819	24 745	22 167	252 471
Target MHCUs < 18 years (1.5% of population)^6^	48 391	47 677	13 747	12 315	140 262
Estimated MHCUs ≥ 18 years	13 270	7200	3766	2547	26 784
Estimated % population covered for MHCUs ≥ 18 years (%)	0.4	0.3	0.5	0.4	0.3
Estimated MHCUs < 18 years	638	570	624	152	1983
Estimated % population covered for MHCUs < 18 years (%)	0.01	0.02	0.07	0.02	0.02

*Sources*: Lund and Flisher 2009; Lund et al. 2009; Statistics South Africa 2012

MHCUs, mental health care users.

### Human resources

The human resources as of March 2015 are summarised in Table 3. There were two vacant posts in addition to those shown; both were psychiatry posts, one in COJ and one in Ekurhuleni. The figures for psychologists, medical and nursing staff reflect those dedicated to the CMHS. For practical purposes general nurses working in the CMHS were included as psychiatric nurses, as they often performed the duties of a psychiatric nurse. In Ekurhuleni, additional nursing staff members were drawn from PHC on an ‘as needed’ basis. The figures for social workers and occupational therapists (including occupational therapy technicians and assistants) reflect those in general PHC to whom CMHS may refer MHCUs. When calculated per 100 000 population, the CMHS human resources in all districts were far below that recommended for minimal service cover of adults.^3^

**TABLE 3:** Human resources serving adult and child and adolescent Community Mental Health Services.

Variable	Norms for adult CMHS only^3^/100 000 population	City of Johannesburg		Ekurhuleni		Sedibeng		West Rand	
		*n*	/100 000	*n*	/100 000	*n*	/100 000	*n*	/100 000
General nurses	9.4	-	-	-	-	-	-	-	-
Psychiatric nurses	3.9	26	0.6	6	0.2	17	1.9	8	1.0
Occupational therapists	3.5	14†	0.3†	13†	0.4†	5†	0.5†	3†	0.4†
OTAs	7.4	-	-	-	-	-	-	-	-
Social workers	6.0	25†	0.6†	17†	0.5†	11†	1.2†	13†	1.6†
Psychologists	2.5	16	0.4	13	0.4	2	0.2	3	0.4
Psychiatrists	0.4	1	0.02	1	0.03	2	0.2	1	0.1
Registrars/medical officers	1.8	8	0.2	5	0.2	5	0.5	3	0.4
Managers	0.5	1	0.02	1	0.03	1	0.1	1	0.1

*Source*: Lund and Flisher 2009

CMHS, Community Mental Health Services; OTA, Occupational Therapy Assistant; PHC, primary health care.

† Serve the whole of PHC as well as CMHS, residential and day care facilities.

### Residential and day care facilities

**FIGURE 2:**
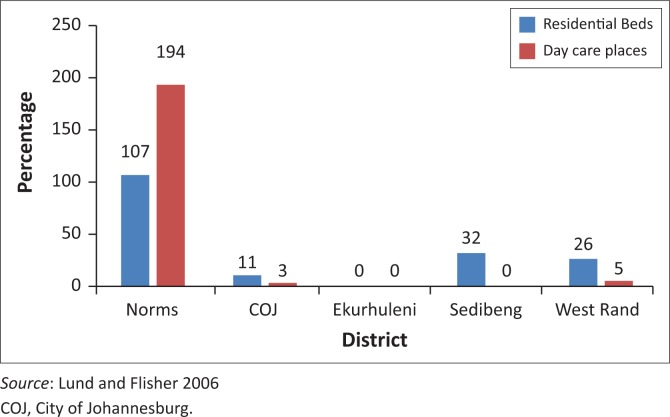
Residential beds and day care places/100 000 population for adult mental health care users in comparison to norms of the Mental Health Policy.

A total of 71 government subsidised community residential homes and day care centres were provided by non-governmental organisations in Southern Gauteng. Forty-six of these were for children and adolescents with intellectual disability. There were no facilities for children and adolescents with mental illness as the primary criterion for admission. There were 25 facilities for adults with mental illness, six of which were day care centres. These were inequitably distributed across Southern Gauteng (Figure 2). They were all run by lay people, and, in general, were appropriate for the provision of a non-restrictive environment and integration into the community. As such, they were most suitable for MHCUs who required a structured home environment but who were willing to comply with medication and refrain from substance use. However, they did not appear suitable for MHCUs who would resist taking medication, present a risk of harm to themselves or others or whose physical frailty necessitates 24-h nursing care.

### Community: Hospital psychiatric staffing ratio

There was a wide variation in the distribution of general hospital-based psychiatrists (Table 4). With the three tertiary academic general hospitals all located in COJ, it had a community: general hospital psychiatrist ratio of 1: 16 (allocating a half-point to part-time consultants). Staffing of the two stand-alone, specialised psychiatric institutions included 15.5 psychiatrists at Sterkfontein hospital and 8.5 at Tara hospital, bringing the overall ratio of government-employed community: hospital-based psychiatrists to 5:44, or roughly 1:9.

**TABLE 4:** Distribution of general hospital-based psychiatrists.

City of Johannesburg		Ekurhuleni		Sedibeng		West Rand	
Hospital	Psychiatrists (*n*)	Hospital	Psychiatrists (*n*)	Hospital	Psychiatrists (*n*)	Hospital	Psychiatrists (*n*)
CMJAH	7	Tembisa	1	Sebokeng	1	Leratong	1
CHBH	7	Natalspruit	1	-	-	-	-
HJH	2	-	-	-	-	-	-

*Source*: Authors’ own work

CMJAH, Charlotte Maxeke Johannesburg Academic Hospital; CHBH, Chris Hani Baragwanath Hospital; HJH, Helen Joseph Hospital.

## Discussion

Overall, the data and information revealed a specialist service which was inappropriately positioned within PHC, a lack of integrated primary mental health care and a very low mental health care coverage of the population. Human resources, residential facilities and day care were far below the numbers recommended by the MH Policy. In addition, there was a marked discrepancy between districts regarding staffing of the CMHS and the balance with general hospital acute psychiatric units.

The positioning of the CMHS as a PHC programme has important potential ramifications. Firstly, the separate administration from the acute hospital units may present a barrier to continuity of care following hospital discharge of MHCUs. Secondly, it might render specialist care too accessible to the community. This may be seen in the high rate of self-referrals and referrals directly from other health and non-health sectors. However, the reasons for people to bypass PHC need further exploration. As no information regarding diagnosis, treatment or disease severity were collected by the DHIS, it cannot be deduced whether these MHCUs had severe illness which warranted specialist care or if they could have been managed at the PHC service level. Thirdly, utilising specialists at PHC level may theoretically contribute to the low rate of integrated primary mental health care, as it could reinforce the misguided impression that all mental illness is to be seen by specialists.

It is probable that incomplete data contributed to the low estimated population coverage because of the routine on-site nature of its collection. However, it is highly unlikely that the coverage was under-estimated by almost 250 000 adults and 140 000 children and adolescents, the target numbers of MHCUs which would be attended to if minimal mental health care coverage was provided (Table 2). Another possibility is that the target figures are an overestimate for South Africa because consensus-based disease prevalence figures were used as local evidence was lacking. The converse though is more likely to be true, as a weighting of only 50% was used for severe disorders^3^ rather than the 80% advocated by the WHO.^14^ In addition, local studies have called for improved CMHS in Gauteng^15,16^ and the low coverage is consistent with the finding by the SASH study that less than 16% of people with common mental illness received any type of treatment within a 12-month period.^13^ There are no national figures for coverage of people with severe mental illness. However, even if the CMHS only treated people with schizophrenia and bipolar disorder, not even 50% were covered.

Two plausible reasons for the low population coverage are the low numbers of mental health visits at PHC and the low allocation of human resources to the CMHS. A large proportion of the population coverage should be for people with mild to moderate illness at PHC.^3^ However, only 0.5% of people attending PHC were seen for mental illness, despite a 12-month prevalence of 16.5% for common mental disorders in the general population of South Africa.^13^ Regarding the effect of specialist human resources on mental health coverage, the highest rate of nursing and medical staffing was in Sedibeng, which also served the largest percentage of the population. This is consistent with other evidence that increased service capacity may increase demand for care.^4^

Not only were there insufficient human resources compared to the numbers recommended in the MH Policy, there were also important changes in the numbers of MHCUs served and the staffing of the CMHS since the 2005 audit.^11^ Overall, the estimated number of adult MHCUs had increased by 24% between 2005 and 2015, and the services had expanded to include child and adolescent mental healthcare. However, the number of dedicated CMHS nurses had been reduced from 31 to 26 in COJ and 31 to 6 in Ekurhuleni over the last 10 years. The numbers of nurses had remained the same in the West Rand. Only in Sedibeng had the nursing staff increased, from 8 to 17. Reasons for this differential pattern in allocating staff to the CMHS between the districts require further exploration in order to inform future planning. One possible cause could be a difference in interpretation of PHC re-engineering, with redeployment of nursing staff from CMHS to integrated PHC in some districts and not others.

Although estimated norms for residential and day care facilities are recommended by the MH policy,^5^ the real extent of unmet need in the community has to be determined. Possible indirect indicators of the need for these facilities could lie in the numbers of homeless and imprisoned mentally ill.

The ratio of one community-based to nine hospital-based psychiatrists is consistent with historical structures of psychiatric care^2^ and current national health plans,^8,9^ yet it is not consistent with cost-effective means of improving access to mental health care.^4^ However, simply rectifying the ratios through redeployment of psychiatrists will very likely have a detrimental effect on the provision of care for the most severely ill. More analysis is required of local needs and corresponding service provision.

### Study limitations

Although using data collected centrally for planning purposes allows for an overview of the entire service, it is also a substantial limitation of the study. Firstly, the data set was not designed for research purposes. This resulted in the use of broad estimates and limited the interpretation of the data, as certain measures, such as diagnostic categories, were not included. Secondly, inaccuracy and incompleteness of data are possible because of the onsite, routine nature of data collection.

## Conclusion

Notwithstanding its limitations, this study is important as it revealed that the existing CMHS in Southern Gauteng did not meet any of the modelled norms referenced in the MH Policy. It highlights the need for further research for the development of CMHS. In addition, it draws attention to the necessity of a comprehensive information system, itself a requirement of the MH Policy.
